# 超高效液相色谱-串联质谱法同时测定人血浆中的塞利尼索、维奈克拉、伏立康唑及泊沙康唑

**DOI:** 10.3724/SP.J.1123.2025.05003

**Published:** 2026-03-08

**Authors:** Xiong XIAO, Yue LI, Jinfang HU, Qing WAN, Hongwei PENG

**Affiliations:** 1.南昌大学第一附属医院药学部，江西 南昌 330000; 1. Department of Pharmacy，The First Affiliated Hospital of Nanchang University，Nanchang 330000，China; 2.南昌大学药学院，江西 南昌 330000; 2. School of Pharmacy，Nanchang University，Nanchang 330000，China

**Keywords:** 超高效液相色谱-串联质谱, 塞利尼索, 维奈克拉, 三唑类抗真菌药, 治疗药物监测, ultra-high performance liquid chromatography-tandem mass spectrometry （UHPLC-MS/MS）, selinexor, venetoclax, triazole antifungal agent, therapeutic drug monitoring

## Abstract

抗血液恶性肿瘤药物常具有治疗窗窄、个体差异显著及药物相互作用风险高等特点，需通过治疗药物监测（TDM）实现个体化给药。对于化疗期间因严重骨髓抑制而引发感染需联合抗真菌治疗的患者，TDM可同时优化抗肿瘤药物与抗真菌药物的血药浓度，在确保抗肿瘤疗效的同时有效控制侵袭性真菌感染风险，这一技术已成为血液系统恶性肿瘤个体化治疗的重要支撑。本研究建立了一种基于超高效液相色谱-串联质谱的分析方法，用于同时测定人血浆中抗肿瘤药物塞利尼索（selinexor， SEL）、维奈克拉（venetoclax， VEN）及抗真菌药物伏立康唑（voriconazole， VOR）、泊沙康唑（posaconazole， POSA）。色谱分离采用Kinetex^®^ XB-C18色谱柱（50 mm×3.0 mm， 2.6 µm），流动相为甲醇-含0.1%甲酸的10 mmol/L乙酸铵溶液，流速0.5 mL/min，梯度洗脱，进样量2 μL，分析时间为4.0 min。采用电喷雾离子源，正离子扫描，多反应监测模式进行测定。本研究对质谱参数进行了优化，并进行了方法学验证，结果表明，该方法线性关系良好，线性相关系数（*r*）均大于0.994，4种药物的线性范围分别为SEL 0.04~1.48 μg/mL、VEN 0.15~5.50 μg/mL、VOR 0.29~11.77 μg/mL和POSA 0.15~6.05 μg/mL；4种药物在各浓度水平的日内精密度和日间精密度相对标准偏差（RSD）≤7.1%，提取回收率均≥85.3%，方法准确度为87.4%~109.0%。采集了30例接受SEL联合VEN治疗的急性髓系白血病患者的81份临床样本进行分析，测得SEL的血药浓度为0.049~0.646 μg/mL，约30%的患者在相同疗程内SEL血药峰浓度（*C*_max_）差异较大。本方法可为临床实践中存在药物相互作用及个体差异的血液系统恶性肿瘤患者的个体化精准治疗提供循证依据。

## Abstract (en)

Drugs used to treat hematologic malignancies often exhibit narrow therapeutic windows， significant inter-individual variability， and high risks of drug-drug interactions， necessitating therapeutic drug monitoring （TDM） for individualized dosing. For patients requiring concomitant antifungal therapy due to infections arising from severe chemotherapy-induced myelosuppression， TDM can optimize plasma concentrations of both antineoplastic and antifungal agents. This concurrent optimization ensures anti-tumor efficacy while effectively mitigating the risk of invasive fungal infections， positioning TDM as a critical component of personalized management in hematologic malignancies. This study established and validated a rapid ultra-high performance liquid chromatography-tandem mass spectrometry （UHPLC-MS/MS） method for the simultaneous quantification of the antineoplastic agents selinexor （SEL） and venetoclax （VEN）， along with the antifungal agents voriconazole （VOR） and posaconazole （POSA） in human plasma. Chromatographic separation was achieved using a Kinetex^®^ XB-C18 column （50 mm × 3.0 mm， 2.6 µm） with a mobile phase consisting of methanol and 10 mmol/L ammonium acetate containing 0.1% formic acid， delivered at a flow rate of 0.5 mL/min under gradient elution conditions. The injection volume was 2 μL， and the total run time was 4.0 min. Detection employed an electrospray ionization source operating in positive ion mode with multiple reaction monitoring （MRM）. Following optimization of mass spectrometric parameters， the method underwent comprehensive validation. Calibration curves demonstrated excellent linearity （*r*>0.994） over the ranges of 0.04-1.48 μg/mL for SEL， 0.15-5.50 μg/mL for VEN， 0.29-11.77 μg/mL for VOR， and 0.15-6.05 μg/mL for POSA. Intra-day and inter-day precisions （RSDs） were ≤7.1% at all concentration levels. Extraction recoveries were ≥85.3%， demonstrating efficient and consistent sample processing. Method accuracy， determined by the percentage deviation of measured concentrations from nominal values， fell within the acceptable range of 87.4% to 109.0% across all QC levels， confirming the method’s trueness. The practical utility of the validated UHPLC-MS/MS method was demonstrated through its application to clinical specimens. We collected and analyzed 81 clinical samples from 30 patients with acute myeloid leukemia treated with the combination of SEL and VEN. Measured plasma concentrations of SEL in these patients ranged from 0.049 μg/mL to 0.646 μg/mL. Notably， significant inter-individual variability in the peak plasma concentration （*C*_max_） of SEL was observed within the same treatment cycle for approximately 30% of the patient cohort. This study offers evidence-based support for personalized precision therapy in this patient population， which exhibits substantial inter-individual variability and complex drug-drug interactions in clinical practice.

多发性骨髓瘤（multiple myeloma， MM）与急性髓系白血病（acute myeloid leukemia， AML）作为临床常见的恶性血液病种，其治疗策略的革新始终是肿瘤学研究的重要方向。近年来，以核输出蛋白（exportin 1， XPO1）为靶点的新型小分子抑制剂塞利尼索（selinexor， SEL）在复发/难治性MM治疗中展现出突破性疗效，并获得美国食品药品监督管理局的临床批准。值得注意的是，XPO1蛋白在AML等恶性肿瘤中的异常高表达特征，促使研究者探索SEL联合B淋巴细胞瘤-2基因（b-cell lymphoma-2，*BCL*-*2*）抑制剂维奈克拉（venetoclax， VEN）的协同治疗方案。据文献［^1^，^2^］报道，该联合用药可通过双重作用机制诱导肿瘤细胞凋亡，然而，药物联用引发的复杂药代动力学相互作用及个体化剂量优化问题，成为制约临床疗效提升的关键问题。

AML患者在强化化疗及靶向治疗过程中，中性粒细胞减少和骨髓抑制等治疗相关并发症的发生显著增加了侵袭性真菌感染风险。根据欧洲白血病感染会议指南^［3］^推荐，三唑类抗真菌药物如伏立康唑（voriconazole， VOR）和泊沙康唑（posaconazole， POSA）已成为侵袭性真菌感染（invasive fungal disease， IFD）预防与治疗的一线选择。然而，此类药物作为强效细胞色素P450 3A4（cytochrome P450 3A4，CYP3A4）酶的抑制剂，可通过药物代谢酶介导的相互作用显著影响VEN（CYP3A4底物）的体内暴露量。据文献［^4^-^7^］报道，VOR与VEN联用可使后者血药浓度-时间曲线下面积增加，可诱发严重不良反应。

基于上述临床挑战，建立精准的治疗药物监测（TDM）体系对确保联合用药安全性至关重要。超高效液相色谱-串联质谱（UHPLC-MS/MS）技术凭借其高灵敏度、卓越的选择性及宽泛的线性范围，已成为复杂基质中多组分同步检测的金标准。但现有文献报道多局限于单一药物检测，缺乏可同步量化SEL、VEN及三唑类抗真菌药的多重分析方法。本研究旨在开发并验证一种高效、可靠的UHPLC-MS/MS方法，实现人血浆中SEL、VEN、VOR和POSA的同步定量分析。

本方法的建立不仅可为临床药物代谢动力学/药物效应动力学（pharmacokinetics/pharmacodynamics， PK/PD）研究提供可靠的技术支持，更有助于构建基于群体药动学模型的个体化给药方案，最终实现疗效优化与不良反应控制的精准平衡。同时有望推动血液肿瘤治疗向精准医学模式转变，为制定个体化治疗决策提供科学依据。

## 1 实验部分

### 1.1 仪器、药品及试剂

AB SCIEX Triple Quad^TM ^4500MD超高效液相色谱-串联质谱仪（美国SCIEX公司）；5810R高速冷冻离心机（德国eppendorf公司）；XW-80A涡旋混合仪（上海琪特分析仪器有限公司）。

SEL、VEN、VEN-d8、VOR、VOR-d3、POSA、POSA-d5等均购自美国TargetMol公司，纯度>99.0%；SEL内标菲达替尼（fedratinib， FED）购自美国MCE公司，纯度99.59%；甲醇及乙腈（质谱纯，中国赛默飞世尔科技有限公司）；无水乙醇、二甲基亚砜（分析纯，国药集团化学试剂有限公司）；甲酸（质谱纯，上海麦克林生化科技股份有限公司）；水为广州屈臣氏蒸馏水。

### 1.2 仪器条件

色谱条件 Kinetex^®^ 2.6 µm XB-C18色谱柱（50 mm×3.0 mm，2.6 μm），流动相A为含0.1%甲酸的10 mmol/L乙酸铵水溶液，流动相B为甲醇，梯度洗脱程序如下：0.01~1.0 min，50%B~90%B；1.0~2.0 min，90%B~100%B；2.0~2.1 min，100%B~50%B；2.1~4.0 min，50%B。流速为0.5 mL/min，柱温为40 ℃，进样量2 μL。

质谱条件 采用电喷雾离子源，正离子模式，多反应监测扫描分析。离子对、去簇电压（declustering potential， DP）、碰撞能（collision energy， CE）及保留时间（*t*_R_）等质谱参数见表 1。

**表1:** 4种药物及其内标的质谱检测参数和保留时间

Compound	Ion pair （*m/z*）	DP/V	CE/eV	*t*_R_/min
Selinexor （SEL）	443.9/334.0	95	30	1.72
Fedratinib （FED）	525.4/97.9	95	67	1.55
Venetoclax （VEN）	868.4/636.3	210	39	2.02
Venetoclax-d8 （VEN-d8）	876.4/329.3	210	58	2.02
Voriconazole （VOR）	350.2/281.2	90	13	1.44
Voriconazole-d3 （VOR-d3）	353.2/284.2	90	13	1.44
Posaconazole （POSA）	701.3/614.4	181	51	1.85
Posaconazole-d5 （POSA-d5）	705.3/618.4	181	51	1.85

DP： declustering potential； CE： collision energy.

### 1.3 血浆样品采集和溶液的配制

#### 1.3.1 样品收集

血浆样本来源于服用SEL联合VEN药物的AML患者，采集自南昌大学第一附属医院血液科。所有样本的收集均在患者签署知情同意书后进行，并得到了医院伦理委员会（IIT【2022】临伦审第129号-1）的批准。全血样本采集后，立即离心分离得到血浆，保存于-80 ℃冰箱。

#### 1.3.2 SEL、VEN、VOR、POSA标准溶液的配制

精密称取SEL 14.78 mg置于棕色容量瓶中，加入无水乙醇溶解并摇匀，配制质量浓度为0.295 6 mg/mL的标准储备液。

精密称取VEN 10.01 mg置于棕色容量瓶中，加入二甲基亚砜溶解并摇匀，配制质量浓度为2.002 mg/mL的标准储备液。

精密称取VOR 11.77 mg置于棕色容量瓶中，加入50%甲醇水溶液溶解并摇匀，配制质量浓度为2.354 mg/mL的标准储备液。

精密称取POSA 15.13 mg置于25 mL容量瓶中，加入甲醇溶解并摇匀，配制质量浓度为0.605 mg/mL的标准储备液。

取适量各标准储备液置于同一容量瓶中，用50%甲醇水溶液稀释，得到SEL、VEN、VOR、POSA质量浓度分别为14.78、55.00、117.70、60.50 μg/mL的混合对照溶液。取混合对照溶液，用50%甲醇水溶液倍比稀释为混合梯度工作液，其中SEL质量浓度为14.78、7.39、3.70、1.85、0.74、0.37 μg/mL；VEN为55.00、27.50、13.75、6.88、2.75、1.38 μg/mL；VOR为117.70、58.85、29.43、14.71、5.89、2.94 μg/mL，POSA为60.50、30.25、15.13、7.56、3.03、1.51 μg/mL。同时用50%甲醇水溶液配制混合质量控制溶液（以下简称质控），SEL高浓度质控（high concentration quality control， H-QC）、中浓度质控（medium concentration quality control， M-QC）、低浓度质控（low concentration quality control， L-QC）的质量浓度分别为11.09、7.39、1.11 μg/mL，VEN的H-QC、M-QC、L-QC为41.25、27.50、4.13 μg/mL，VOR的H-QC、M-QC、L-QC为88.28、58.85、8.83 μg/mL，POSA的H-QC、M-QC、L-QC为45.38、30.25、4.54 μg/mL。所有溶液-20 ℃避光保存。

#### 1.3.3 内标工作液的配制

精密称取 POSA-d5、VEN-d8、VOR-d3及FED各1.0 mg，加入适量二甲基亚砜溶解，用含0.1%甲酸的甲醇稀释成质量浓度均为1.0 μg/mL的混合内标工作液，-20 ℃避光保存备用。SEL药物暂无市售商品化的同位素内标，本研究参考文献［^8^］采用FED为内标，后续的方法学验证结果显示，FED可作为SEL内标用于其血药浓度监测。

#### 1.3.4 血浆基质匹配混合梯度工作液和血浆基质匹配质控溶液的配制

取9份900 μL空白人血浆于1.5 mL离心管中（编号1~9），向编号为1~6的离心管中分别加入1.3.2节系列质量浓度的混合梯度工作液（各100 μL）。制得4种药物的血浆基质匹配混合梯度工作液；向编号为7~9的离心管中分别加入1.3.2节3个浓度水平的质控工作溶液（各100 μL），制得4种药物的基质匹配质控溶液。

#### 1.3.5 血浆样品预处理

取100 μL血浆样品，加入400 μL样本释放剂（含0.1%甲酸的乙腈）和20 μL内标工作液，涡旋5 min，4 ℃下以13 000 r/min离心10 min。取200 μL上清液，待进样分析。

## 2 结果与讨论

### 2.1 色谱条件的优化

为了获得4种药物的最佳电离效果和色谱峰形，考虑到使用质谱正离子模式进行分析时，4种药物在电离过程中需提供足够的质子，参考文献［^9^，^10^］，分别向甲醇-水和乙腈-水的基础流动相体系中添加10 mmol/L乙酸铵缓冲盐、0.1%甲酸。结果显示：乙腈-水和甲醇-水作为流动相时，4种药物色谱峰形较差。在流动相体系中分别添加10 mmol/L乙酸铵缓冲盐，色谱峰形得到一定改善，但大部分药物响应仍较低。在流动相体系中添加0.1%甲酸后，目标化合物响应提高。对比含0.1%甲酸的10 mmol/L乙酸铵水溶液-甲醇体系和0.1%甲酸10 mmol/L乙酸铵水溶液-乙腈体系，结果发现，有机相为乙腈时4种药物的峰形和响应与甲醇体系无明显差异，考虑到试剂的成本及毒性，故选择含0.1%甲酸的10 mmol/L乙酸铵水溶液-甲醇为流动相。本研究中4种药物的极性均较小，为提高质谱分析效率，故设置流动相洗脱梯度程序初始有机相比例为50%，后梯度升高有机相比例，使得4种药物能快速分离。同时50%有机相比例与血浆样本蛋白沉淀后溶液有机相比例接近，可避免溶剂效应导致峰形异常。

### 2.2 质谱条件的优化

将质谱仪激活到Mass only状态，分别在其正离子和负离子模式下，使用针泵持续注射质量浓度为500 ng/mL的单个药物溶液至质谱仪中。发现4种药物在质谱正离子模式下灵敏度最佳，因此选择在该模式下对*m/z* 300~880范围内的离子进行全扫描，确定每种药物的母离子，再进行子离子扫描，每种药物选取响应值高的特征离子对为定量离子对。进一步对DP、CE值进行优化，选择药物质谱响应最佳的参数，初步建立质谱方法。

之后，质谱仪切换到液相色谱-质谱联用状态，采用1.3.5节下前处理方法处理血浆基质匹配混合梯度工作液样本（质量浓度分别为SEL 1.48 μg/mL、VEN 5.50 μg/mL、VOR 11.77 μg/mL和POSA 6.05 μg/mL），进样分析。同时不断调整质谱DP、CE、离子源温度、气流量及进样体积的参数，以4种药物质谱离子响应为评价指标，优化出最佳质谱参数，优化结果见表1。

### 2.3 样品前处理条件的优化

本研究采用蛋白沉淀方式进行血浆样品前处理。在对人血浆中的多种药物进行同时定量检测时，蛋白沉淀剂的选择至关重要。需考虑各种药物在有机溶剂中的溶解度以及溶剂对血浆基质的沉淀效率等因素。本研究参考已有报道^［11，12］^，选择极性溶剂乙腈作为蛋白沉淀剂，其对血浆中蛋白的沉淀效率高，并且能够有效减少对样品中磷脂及脂肪的过多提取，降低基质效应。本研究实验过程中，通过对比1∶2、1∶4、1∶8、1∶10等比例的血浆-有机溶剂体积比处理血浆基质匹配混合梯度工作液样本（质量浓度分别为SEL 1.48 μg/mL、VEN 5.50 μg/mL、VOR 11.77 μg/mL和POSA 6.05 μg/mL）的质谱响应值，发现血浆-有机溶剂体积比为1∶4是最佳蛋白沉淀体积比。

### 2.4 方法学验证

#### 2.4.1 选择性

为验证该分析方法能够区分待测药物、内标、基质的内源性物质和样品中的其他物质，本研究取6个来自不同健康人的血浆样本100 μL，加入20 μL含0.1%甲酸的甲醇替代内标工作液，按照1.3.5节下血浆样品预处理方法处理，进样检测，获得6份空白血浆样品色谱图。另取空白血浆样本100 μL，按照1.3.5节下血浆样品预处理方法，进样检测，获得仅含内标的空白血浆样品色谱图。再另取上述空白血浆90 μL加入10 μL混合梯度工作液（质量浓度分别为SEL 1.48 μg/mL、VEN 5.50 μg/mL、VOR 11.77 μg/mL和POSA 6.05 μg/mL）配成血浆基质匹配混合梯度工作液样品。按照1.3.5节下血浆样品预处理方法处理，进样检测，获得含待测药物及内标的血浆样品色谱图。通过分析上述所获得的色谱图发现，SEL、VEN、VOR和POSA的保留时间分别为2.02、1.72、1.44、1.85 min。6个空白血浆样品在各待测药物离子检测通道相对应保留时间处均未出现色谱峰，仅含内标的空白血浆样品在各待测药物离子检测通道相对应保留时间处也均未出现色谱峰，表明该分析方法能够很好地区分待测药物、内标、基质的内源性物质和样品中的其他物质，方法选择性良好。其中一位健康人空白血浆样品色谱图与血浆基质匹配混合梯度工作液样品（加标血浆）中各待测药物的典型色谱图见图1。

**图1:**
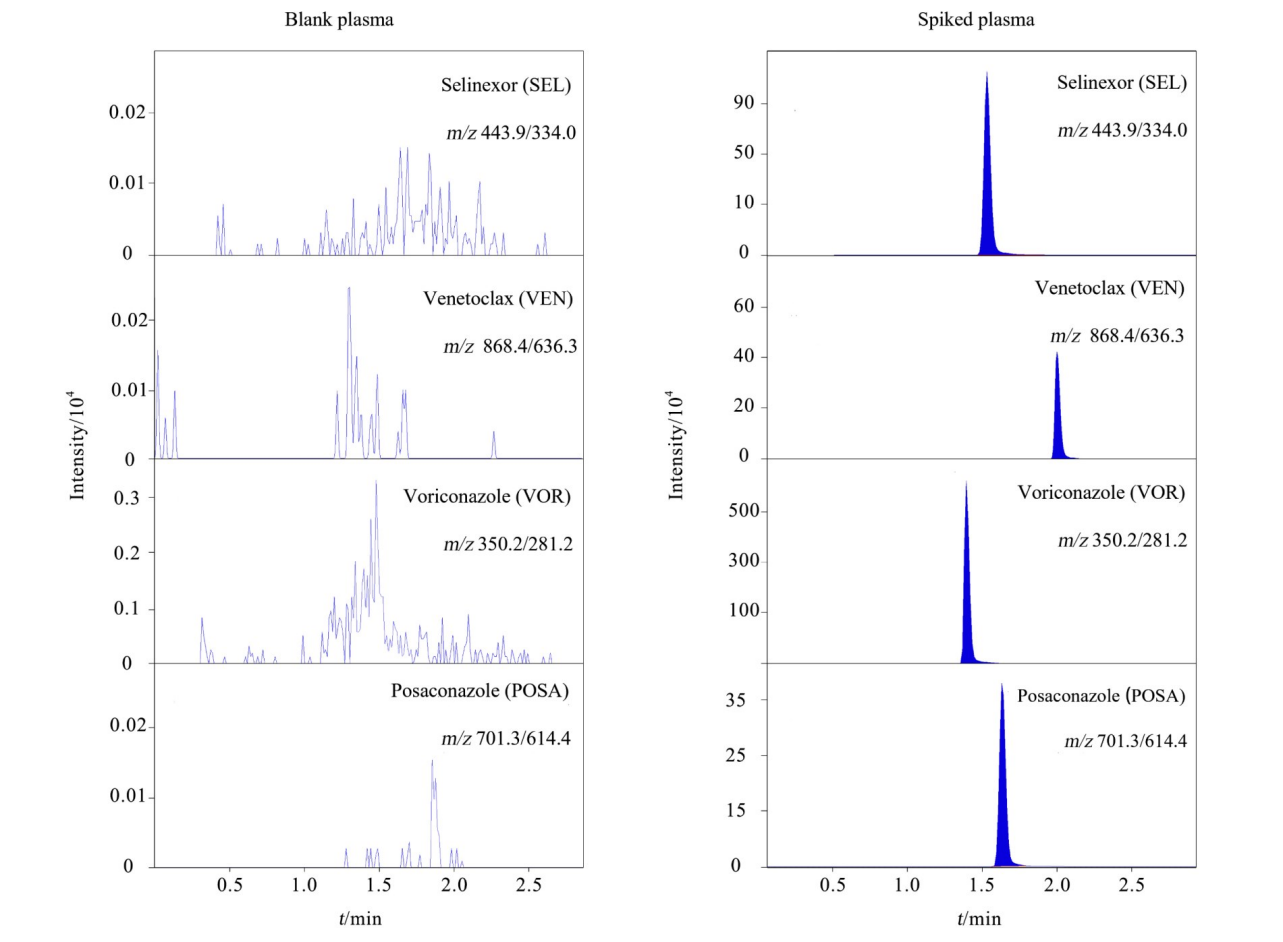
健康人空白血浆样品与血浆基质匹配混合梯度工作液样品（加标血浆）中各待测药物的典型色谱图

#### 2.4.2 基质效应

取6个不同健康人的空白血浆90 μL，按1.3.5节血浆样品处理方法处理得到不含待测药物的空白基质，加入L-QC、H-QC两个浓度水平的混合质量控制溶液10 μL，制备血浆基质样品溶液；用去离子水代替血浆，加入L-QC、H-QC两个浓度水平的混合质量控制溶液10 μL，按1.3.5节血浆样品处理方法处理得到无基质样品溶液，进样测定。分别计算血浆基质样品溶液和无基质样品溶液中待测药物峰面积与其内标峰面积的比值，以血浆基质样品溶液待测药物峰面积与其内标峰面积比值除以无基质样品溶液待测药物峰面积与其内标峰面积比值计算内标归一化基质效应因子，然后计算其RSD^［13］^。结果表明，SEL在L-QC和H-QC两个浓度水平下的内标归一化基质因子的RSD值分别为8.83%和2.38%，VEN分别为2.80%和1.25%，VOR分别为4.10%和2.18%，POSA分别为4.34%和4.12%，表明上述4种药物的测定不受基质效应的影响。

#### 2.4.3 标准曲线和定量限

取1.3.4节下血浆基质匹配混合溶液和血浆基质匹配质控溶液，按1.3.5节血浆样品前处理方法处理，进样分析。分别以待测物与对应内标峰面积比值*（Y）*对 SEL、VEN、VOR、POSA质量浓度（*X*）作回归分析，权重为1/*X*。在满足准确度和精密度要求下，结合临床实际应用，确定定量限（limit of quantification，LOQ）。结果表明，SEL的回归方程为*Y*=16.26*X*+0.064（*r*=0.996），线性范围为0.04～1.48 μg/mL，定量限为0.04 μg/mL；VEN的回归方程为*Y*=4.88*X*+0.096（*r*=0.998），线性范围为 0.14～5.50 μg/mL，定量限为0.14 μg/mL；VOR的回归方程为*Y*=6.48*X*+0.31（*r*=0.998），线性范围为0.29～11.77 μg/mL，定量限为0.29 μg/mL；POSA的回归方程为*Y*=10.49*X*+0.23（*r*=0.999），线性范围为0.15～6.05 μg/mL，定量限为0.15 μg/mL。

#### 2.4.4 精密度、准确度及提取率

分别配制 SEL、VEN、VOR、POSA 的LOQ、L-QC、M-QC、H-QC 4个浓度水平的血浆基质匹配质控溶液，每个浓度平行制备5份，按照1.3.5节下血浆样品前处理方法处理，进样测定，记录峰面积。同样方法连续3天制备3批1.3.4节下血浆基质匹配混合溶液和血浆基质匹配质控溶液并进样分析，计算日内及日间精密度（RSD）和准确度。同时配制 SEL、VEN、VOR、POSA 的L-QC、M-QC、H-QC 3个浓度水平的血浆基质匹配质控溶液各6份，其中3份于提取前加入样本释放剂，提取后加入内标，另3份于提取后加入样本释放剂和内标。进样分析，计算各个药物的提取率。结果见表2，数据表明，4种药物的日内、日间精密度均不超过7.1%，准确度为87.2%～107.3%，提取回收率为85.3%～97.5%，符合生物样本分析的相关法规^［14］^要求。

**表2:** 4种药物在LOQ、L-QC、M-QC和H-QC水平下的日内精密度、日间精密度（*n=*5）及在L-QC、M-QC和H-QC水平下的提取回收率（*n=*6）

Analyte	Content/ （μg/mL）	Intra-day RSD/%	Inter-day RSD/%	Extraction recovery/%
Selinexor	0.0	5.0	3.8	/
	0.1	5.3	5.7	87.9
	0.7	5.9	6.6	90.3
	1.1	3.5	3.1	96.1
Venetoclax	0.1	5.7	6.4	/
	0.4	3.6	4.2	85.3
	2.8	0.3	0.4	87.5
	4.1	4.3	2.6	89.3
Voriconazole	0.3	1.9	0.7	/
	0.9	4.6	1.3	93.1
	5.9	4.1	1.0	92.8
	8.8	4.6	1.9	96.1
Posaconazole	0.2	0.6	1.5	/
	0.5	7.1	2.7	88.0
	3.0	1.0	1.3	91.9
	4.5	3.0	3.2	97.5

L-QC： low concentration quality control； M-QC： medium concentration quality control； H-QC： high concentration quality control. /： the experiment was not conducted.

#### 2.4.5 稳定性

取L-QC、H-QC浓度水平的血浆基质匹配质控溶液，每个浓度平行3份，考察前处理后溶液在进样器（10 ℃）放置 24 h的稳定性以及血浆样品在4 ℃放置 48 h、-80 ℃冻融循环3次和-80 ℃放置30 d等的稳定性，计算各药物的RSD。结果表明，各待测药物在L-QC、H-QC水平下，测定结果均值与标示浓度的偏差均在±15%范围内，每个浓度平行测定结果的RSD值均≤6.43（见表3），表明待测药物在上述条件下的稳定性良好。

**表3:** 低、高2个浓度水平下4种药物的血浆基质匹配质控溶液及血浆前处理后溶液放于指定温度环境时的稳定性（*n*=3）

Analyte	Content/ （μg/mL）	RSDs/%			
		Freeze and thaw	-80 ℃	4 ℃	Auto-sampler
Selinexor	0.11	3.64	6.43	0.58	4.59
	1.11	2.30	5.27	1.55	3.54
Venetoclax	0.41	3.35	3.35	3.89	3.61
	4.13	3.77	3.77	2.65	1.28
Voriconazole	0.88	0.67	1.07	2.06	4.56
	8.83	2.24	2.33	1.23	3.35
Posaconazole	0.45	2.17	3.35	5.13	2.45
	4.54	2.96	3.43	2.64	2.87

#### 2.4.6 稀释完整性

取1.3.2节下标准储备液，用50%甲醇水溶液稀释最高浓度水平溶液5倍、3倍、2倍。取10 μL，加入空白人血浆90 μL，涡旋1 min，制成高浓度血浆基质匹配溶液，并用空白血浆稀释5倍、3倍、2倍（*n*=5），测定准确度和精密度。结果表明，准确度为89.4%～105.2%，精密度（批内变异系数）均小于15%，说明该方法稀释结果准确可靠。

#### 2.4.7 携带污染

进样1.3.5节方法处理后的血浆基质匹配混合梯度工作液样本（SEL 1.48 μg/mL、VEN 5.50 μg/mL、VOR 11.77 μg/mL和POSA 6.05 μg/mL），然后进样1.3.5节下前处理方法处理后的空白血浆样品，将空白血浆样品中的残留响应值和定量限血浆样本及仅加内标血浆样本响应值进行比较。结果表明，空白血浆样品中的残留响应低于定量限的20%，并且低于内标的5%，表明残留不影响4种待测药物的准确定量。

### 2.5 临床样本血药浓度检测结果分析

#### 2.5.1 VEN血药浓度监测

本课题组前期监测了56例服用VEN联合VOR、POSA药物的白血病患者的血药浓度。分析数据发现，VOR、POSA可能通过抑制CYP3A4肝药酶活性从而影响VEN药物代谢，VEN血药浓度升高可能会导致VEN药物不良反应发生率增加，患者遭受不良反应伤害风险增加，相关研究成果已发表在国际期刊上^［15］^。而VOR、POSA药物药代动力学个体差异大，血药浓度受年龄、基因、肝肾功能等因素影响。本研究建立的方法可同时对上述药物进行血药浓度监测，有助于及时调整剂量，确保有效浓度，避免浓度不足导致治疗失败或浓度过高引发毒性。

#### 2.5.2 SEL血药浓度监测

本研究收集了30位服用SEL联合VEN药物的AML患者的血药浓度数据，30位服用SEL联合VEN的AML患者，每周固定时间服药2次，于服药后4 h采血监测SEL峰浓度（*C*_max_），共计收集81份样本，其中合用VOR有22次，合用POSA有5次。将单用SEL组的*C*_max_与联用三唑类药物的各组*C*_max_进行独立样本T检验，联用POSA组的SEL的*C*_max_小于单用SEL组，且具有统计学意义，详见图2。但局限于样本量小的原因，该结论可能需要后续更大样本论证。

**图2:**
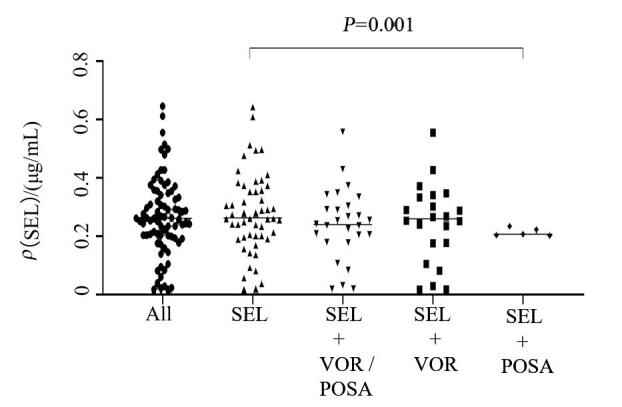
SEL *C*_max_独立样本T检验分析结果

本研究取服药后4 h浓度为*C*_max_^［16，17］^。监测了30位患者总计81份样本， 30%的患者在服用相同剂量的SEL药物后，其*C*_max_值出现27.29%~71.43%的波动，部分数据见表4。通过分析患者病历数据，未发现SEL *C*_max_值与血细胞分析相关指标及肝肾功能的相关性。对于*C*_max_值出现较大差异的原因，初步考虑是患者病情发生变化，如感染、低蛋白血症等因素导致药物PK/PD参数的变化，此问题将在后续的研究中继续关注。现阶段暂无明确的SEL联合VEN治疗AML的药物治疗浓度窗，故推荐临床治疗过程中，通过观察临床患者的治疗效果及出现的不良反应，采用本研究所建立方法进行药物浓度监测，依据药物浓度与患者临床表现及时调整治疗方案。

**表4:** 部分患者同一治疗周期多次血药浓度监测的SEL *C*_max_值 (μg/mL)

Blood collection time	Patients				
	#1	#2	#3	#4	#5
Week 1， 1st day， C4h^*^	0.252	0.376	0.049	0.355	0.347
Week 1， 5th day， C4h	0.047	0.479	0.288	0.646	0.515
Week 2， 1st day， C4h	0.427	0.095	0.105	0.276	0.612
Week 2， 5th day， C4h	0.263	0.326			
Week 3， 1st day， C4h	0.084	0.243			

* Blood concentration at 4 h post-dose on the first day of Week 1.

### 2.6 与已有方法比较

现有报道均是关于这4种药物中的1种药物或两种药物检测^［12，18］^，日常检测过程中需要单独对每种药物建立标准曲线，耗时耗力，检测效率低下，不能快速反馈检测结果至临床，对于临床治疗帮助有限。而本方法可以同时检测样品中的SEL、VEN、VOR、POSA，缩短了报告发送时间，便于在患者出现治疗效果不佳或出现药物毒性反应时及时调整药物治疗方案。

## 3 结论

本研究建立了使用超高效液相色谱-串联质谱同时测定人血浆中SEL、POSA、VEN及VOR的方法。该法所需要的样本量少，选择性好，灵敏度高，准确稳定，符合临床高通量、快速检测的需求，适用于临床MM与AML患者药物浓度的监测。
